# The Real-World Clinical Impact of Plasma mNGS Testing: an Observational Study

**DOI:** 10.1128/spectrum.03983-22

**Published:** 2023-03-22

**Authors:** Dongsheng Han, Fei Yu, Dan Zhang, Qing Yang, Mengxiao Xie, Lingjun Yuan, Jieyuan Zheng, Jingchao Wang, Jieting Zhou, Yanyan Xiao, Shufa Zheng, Yu Chen

**Affiliations:** a Department of Laboratory Medicine, the First Affiliated Hospital, Zhejiang University school of Medicine, Hangzhou, Zhejiang, People's Republic of China; b Key Laboratory of Clinical In Vitro Diagnostic Techniques of Zhejiang Province, Hangzhou, Zhejiang, People's Republic of China; c Institute of Laboratory Medicine, Zhejiang University, Hangzhou, Zhejiang, People's Republic of China; Hospital Saint-Louis

**Keywords:** infectious disease, metagenomic next-generation sequencing, pathogen, plasma

## Abstract

Plasma metagenomic next-generation sequencing (mNGS) testing is a promising diagnostic modality for infectious diseases, but its real-world clinical impact is poorly understood. We reviewed patients who had undergone plasma mNGS at a general hospital to evaluate the clinical utility of plasma mNGS testing. A total of 76.9% (113/147) of plasma mNGS tests had a positive result. A total of 196 microorganisms (58) were identified and reported, of which 75.6% (148/196) were clinically relevant. The median stringent mapped read number (SMRN) of clinically relevant organisms was 88 versus 22 for irrelevant organisms (*P* = 0.04). Based on the clinically adjudicated diagnosis, the positive and negative percent agreements of plasma mNGS testing for identifying a clinically defined infection were 95.2% and 67.4%, respectively. The plasma mNGS results led to a positive impact in 83 (57.1%) patients by diagnosing or ruling out infection and initiating targeted therapy. However, only 32.4% (11/34) of negative mNGS tests showed a positive impact, suggesting that plasma mNGS testing alone may not be a powerful tool to rule out infection in clinical practice. In the subset of 37 patients positive for both plasma mNGS and conventional testing, mNGS identified the pathogen(s) 2 days (IQR = 0.75 to 4.25) earlier than conventional testing. mNGS enables pathogen identification within 24 h, but given that the detection of clinically irrelevant organisms and nearly half of the tests result in no or a negative clinical impact, more clinical practice and studies are required to better understand who and when to test and how to optimally integrate mNGS into the infectious disease diagnostic workup.

**IMPORTANCE** In this study, we show that although plasma mNGS testing significantly improved the detection rate of tested samples, nearly one in four (24.5%, 48/196) mNGS tests reported organisms were not clinically relevant, emphasizing the importance of cautious interpretation and infectious disease consultation. Moreover, based on clinical adjudication, plasma mNGS testing resulted in no or a negative impact in nearly half (43.5%, 64/147) of patients in the current study, indicating that how best to integrate this advanced method into current infectious disease diagnostic frameworks to maximize its clinical utility in real-world practice is an important question. Therefore, recommending plasma mNGS testing as a routine supplement to first-line diagnostic tests for infectious diseases faces great challenges. The decision to conduct mNGS testing should take into account the diagnostic performance, turnaround time and cost-effectiveness of mNGS, as well as the availability of conventional tests.

## INTRODUCTION

Infectious diseases remain a global public health concern. Significantly greater numbers remain undiagnosed, both in the inpatient and community settings, resulting in substantial morbidity and mortality (1). Critical and timely intervention for infectious disease relies on rapid and accurate pathogen identification. Metagenomic next-generation sequencing (mNGS) of pathogen nucleic acids in clinical samples has emerged as a promising “one-size-fits-all” approach for hypothesis-free diagnosis of potentially all infectious pathogens in a relatively short time frame (approximately a day or less) (2). Over the past few years, an ever-increasing number of studies have demonstrated that the use of mNGS technology to detect cell-free nucleic acids (cfNA) in plasma (i.e., plasma mNGS testing) can achieve rapid, noninvasive, and unbiased identification of pathogens, and supported the potential relevance of using this advanced technology in a routine setting (3, 4). However, the operation procedure of current plasma mNGS testing (involving sampling, cfNA isolation, library preparation, sequencing, bioinformatics analysis, and interpretation of results) is complex and the cost of testing is high (4), emphasizing the importance of improving our understanding of the optimal timing of adoption, the appropriate testing patients and the enhanced performance of this advanced assay compared with standard of care microbiological diagnostics in real-world practice. To our knowledge, only a few studies have retrospectively evaluated the clinical utility of microbial cfDNA mNGS assays (5–8), all using the Karius test (Karius, Inc., Redwood City, CA, USA), which does not detect RNA viruses (3). However, each study assigned its own criteria, resulting in significant variation in assessments of clinical impact in real-world applications (9–11). The actual clinical impact on patient management of plasma mNGS tests targeting both DNA pathogens and RNA viruses in real-world clinical practice remains to be assessed.

In the clinical laboratory of our hospital, we have established a sequencing platform for mNGS testing based on a commercial solution (http://www.visionmedicals.com/) (12–15) to provide patients with a real-time sequencing service within the hospital. The whole protocol for detecting both cell-free DNA and cell-free RNA in blood samples include a wet lab workflow (i.e., plasma isolation, cell-free nucleic acid extraction, library preparation, sequencing) and a dry lab workflow (i.e., bioinformatics analysis and interpretation of results) (see supplementary methods). Prior to testing, a quantified unique molecular sample identifier (UMSI) was added to each specimen as an identity and internal control to monitor for possible cross-contamination and false negatives. DNA UMSIs are PCR products derived from unique nucleic acid fragments of the genome of Oryza sativa with a length of 400 to 600 bp. RNA UMSIs were transcription products using T7 RNA polymerase and DNase for digestion depleted DNA. Up to 20 libraries were processed in parallel in each sequencing run. At our hospital, clinicians have postulated that plasma mNGS may be useful in the following clinical scenarios: (i) rapid and definitive diagnosis is urgently needed in critically ill patients; (ii) suspected infection but etiological evidence that cannot be obtained by conventional microbiological testing methods; (iii) deep-seated and difficult-to-sample infections, such as invasive fungal infections, pneumonia, or deep-seated abscesses; (iv) useful invasive samples that cannot be obtained; and (v) possible secondary or occult infections can be monitored after solid organ transplantation. The aims of this study were to evaluate the test performance characteristics of plasma mNGS implemented in our hospital and to discuss how mNGS findings affect clinical management.

## RESULTS

### Patient characteristics.

A total of 147 patients who underwent plasma mNGS testing at the First Affiliated Hospital, Zhejiang University School of Medicine (FAHZU) were enrolled in the present study (Table 1). Forty-three patients (29.3%) were admitted to the intensive care unit (ICU). There were 96 (65.3%) males and 94 (63.9%) immunocompromised patients, most commonly hematological diseases (29.9%), solid-organ transplant (18.4%), and organ dysfunction (14.3%) (Table 1). A total of 139 (94.6%) had received empirical antimicrobial therapy. Concern for fever of unknown origin was the most common indication for testing (32.7% of all tests), followed by suspected pulmonary infection (19.7%) and sepsis (18.4%). A subset of seven patients with symptoms such as lung changes on chest CT, abdominal pain, or pleural effusion used mNGS tests for ruling out infection due to unavailability of primary lesion samples for analysis at that time. Four individuals underwent mNGS tests for monitoring possible secondary or occult infections after heart (*n* = 1), liver (*n* = 1), or kidney (*n* = 2) transplantation.

**TABLE 1:** Demographic and clinical characteristics of patients included in this study^*a*^

The study cohort	Number of patients, *n* (%)	Blood culture with a positive result, *n* (%)	Conventional microbiological tests (culture, serology, and/or PCR) with a positive result, *n* (%)	Plasma mNGS testing with a positive result, *n* (%)	Plasma mNGS tests with Positive clinical impact, *n* (%)
No. of patients	147 (100)	30 (20.4)	84 (57.1)	113 (76.9)	83 (20.4)
Mean age (SD)	50.7 (17.7)				
Male sex	96 (65.3)				
Primary medical condition					
Solid-organ transplant	27 (18.4)	11 (40.7)	22 (81.5)	24 (88.9)	12 (44.4)
Hematological	44 (29.9)	12 (27.3)	26 (59.1)	34 (77.3)	28 (63.6)
HSCT	15 (10.2)	6 (40)	10 (66.7)	14 (93.3)	11 (73.3)
Leukemia without HSCT	22 (15)	5 (22.7)	12 (54.5)	16 (72.7)	15 (68.2)
Lymphoma without HSCT	6 (4.1)	1 (16.7)	4 (66.7)	4 (66.7)	1 (16.7)
MDS	1 (0.7)	0 (0)	0 (0)	1 (100)	1 (100)
Cancer	7 (4.8)	2 (28.6)	5 (71.4)	4 (57.1)	3 (42.9)
Organ dysfunction	22 (15)	1 (4.5)	8 (36.4)	15 (68.2)	7 (31.8)
Cardiovascular diseases	8 (5.4)	1 (12.5)	3 (37.5)	4 (50)	4 (50)
Trauma	5 (3.4)	0 (0)	3 (60)	3 (60)	2 (40)
Autoimmune disease	7 (4.8)	0 (0)	4 (57.1)	4 (57.1)	4 (57.1)
HIV	5 (3.4)	1 (20)	5 (100)	5 (100)	5 (100)
Diabetes	2 (1.4)	1 (50)	2 (100)	2 (100)	2 (100)
No underlying medical condition	20 (13.6)	1 (5)	6 (30)	17 (85)	16 (80)
Immunocompromised					
Yes	94 (63.9)	24 (25.5)	62 (66)	73 (77.7)	52 (55.3)
No	53 (36.1)	6 (11.3)	22 (41.5)	40 (75.5)	31 (58.5)
Antibiotic therapy prior to mNGS testing					
Yes	139 (94.6)	28 (20.1)	80 (57.6)	108 (77.7)	81 (58.3)
No	8 (5.4)	2 (25)	4 (50)	5 (62.5)	2 (25)
The indication for mNGS test					
FUO	48 (32.7)	11 (22.9)	24 (50)	37 (77.1)	27 (56.3)
PI	29 (19.7)	3 (10.3)	22 (75.9)	27 (93.1)	21 (72.4)
Sepsis	27 (18.4)	10 (37)	18 (66.7)	22 (81.5)	12 (44.4)
SBI without sepsis	14 (9.5)	4 (28.6)	4 (28.6)	9 (64.3)	8 (57.1)
Suspected AI	6 (4.1)	0 (0)	3 (50)	4 (66.7)	2 (33.3)
IE	4 (2.7)	0 (0)	2 (50)	2 (50)	2 (50)
Suspected UTI	1 (0.7)	1 (100)	1 (100)	1 (100)	0 (0)
Suspected CNSI	1 (0.7)	0 (0)	0 (0)	1 (100)	1 (100)
Abscess	6 (4.1)	1 (16.7)	4 (66.7)	4 (66.7)	4 (66.7)
Rule-out infection	7 (4.8)	0 (0)	0 (0)	3 (42.9)	5 (71.4)
Monitoring	4 (2.7)	0 (0)	4 (100)	3 (75)	1 (25)

a HSCT, hematopoietic stem cell transplant; MDS, myelodysplastic syndrome; FUO, fever of unknown origin; PI, suspected pulmonary infection; SBI, suspected bloodstream infection; AI, abdominal infection; IE, infective endocarditis; UTI, urinary tract infection; CNSI, central nervous system infection.

### Diagnostic performance of plasma mNGS testing.

**FIG 1:**
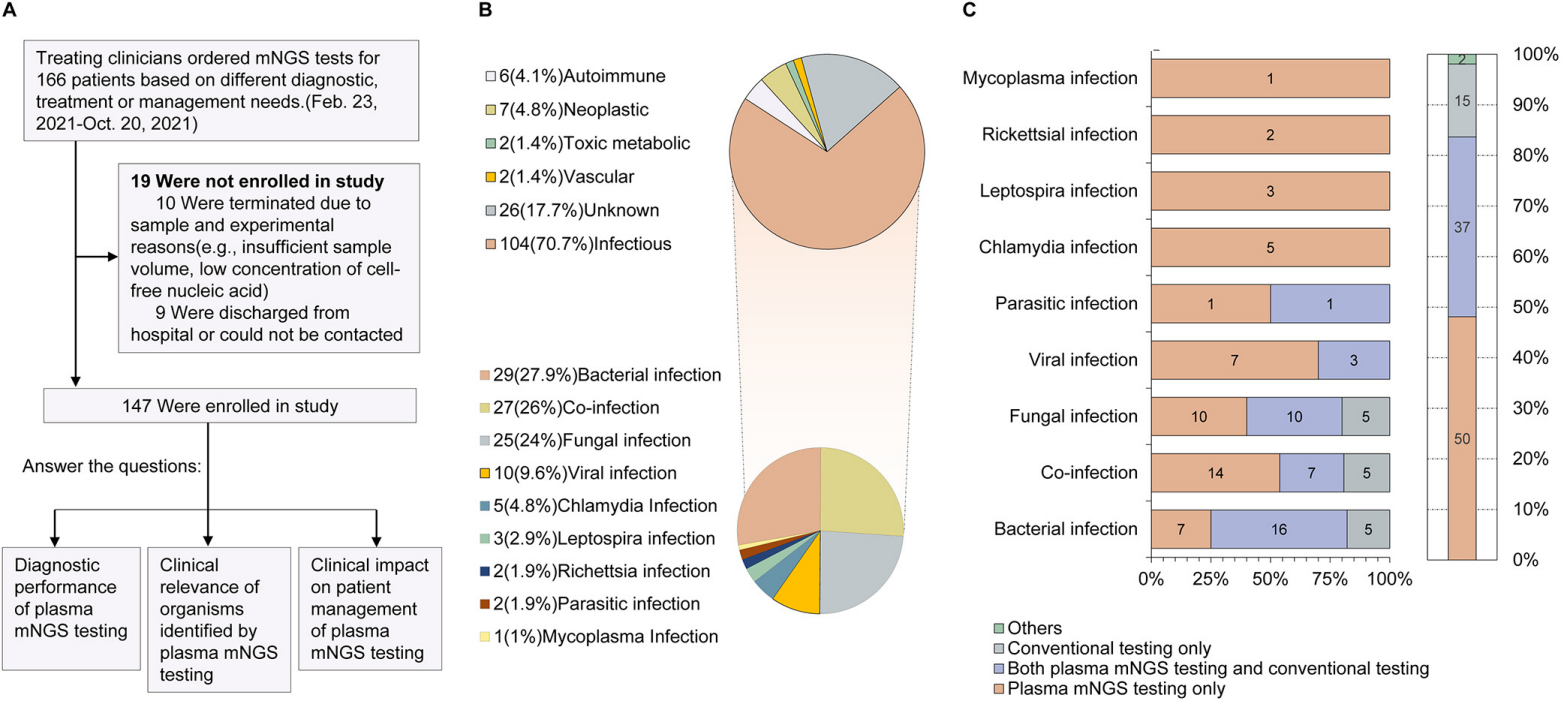
Contribution of plasma mNGS testing in the diagnosis of the enrolled patients. (A) The enrolled patients and research questions. (B) Diagnoses of the enrolled patients were established by the clinical treatment team based on all laboratory data, radiology results, clinical manifestations, treatment, and disease outcomes. (C) Contribution of plasma mNGS testing in identifying true pathogens for the diagnosed infectious patients. Others included one bacterial infection diagnosed by abdominal drainage mNGS and one coinfection diagnosed by plasma mNGS, conventional testing and bronchoalveolar lavage fluid (BALF) mNGS.

Based on a composite reference standard that combined results from microbiological tests (including cultures, serology, and PCR) performed within 7 days of presentation and clinical adjudication made by the treating team, an etiologic diagnosis was identified by conventional testing in 81.6% (*n* = 121) of the study patients, with infectious (*n* = 104, 70.7%) as the most common diagnostic category (Fig. 1B). A total of 48.1% (*n* = 50) of the 104 infections were identified with plasma mNGS alone (Fig. 1C), including 25% (7/28) of bacterial infections, 40% (10/25) of fungal infections, 53.8% (14/26) of coinfections, 70% (7/10) of viral infections, 50% (1/2) of parasite infections and all chlamydia (*n* = 5), Leptospira (*n* = 3), rickettsial (*n* = 2), and mycoplasma (*n* = 1) infections. The positive and negative percent agreements (PPA and NPA) of plasma mNGS by test sent were 95.2% (95% confidence interval [CI] = 88.6% to 98.2%) and 67.4% (95% CI = 51.3% to 80.5%), respectively, the proportion of true positives out of mNGS positives was 87.6% (95% CI = 79.7% to 92.8%), and the proportion of true negatives out of negative mNGS tests was 85.3% (95% CI = 68.2% to 94.5%) (Table 2).

**TABLE 2:** Diagnostic performance of plasma mNGS testing compared to a composite reference standard^*a*^

Plasma mNGS testing	The etiologic diagnosis of the study patients (*n* = 147)		Diagnostic performance
	Infectious (*n* = 104)	Others (*n* = 43)	
Positive plasma mNGS test (*n* = 113)	True positive (TP): 99	False positive (FP): 14	True positives out of mNGS Positives: 87.6%, 95% CI = 79.7 to 92.8%
Negative plasma mNGS tests (*n* = 34)	False negative (FN): 5	True negative (TN): 29	True negatives out of mNGS negatives: 85.3%, 95% CI = 68.2 to 94.5%
Diagnostic performance	Positive percent agreement: 95.2%, 95% CI = 88.6 to 98.2%	Negative percent agreement: 85.3%, 95% CI = 68.2 to 94.5%	

a The composite reference standard combined the results from all microbiological tests from cultures, serology and PCR performed within seven days of presentation and clinical adjudication made by treating team.

### Clinical relevance of organisms identified with mNGS.

In this study, a pathogen was identified by blood culture in only 30 (20.4%) of the 147 patients, most of whom had undergone solid organ transplantation or had hematological disorders (Table 1). A total of 84 (57.1%) patients had one or more possible pathogens detected by the combination of conventional microbiological tests (culture, serology, and/or PCR). Compared with conventional tests, the plasma mNGS testing provided positive results for 113 (76.9%) patients, indicating that mNGS testing can greatly improve the detection rate.

**FIG 2:**
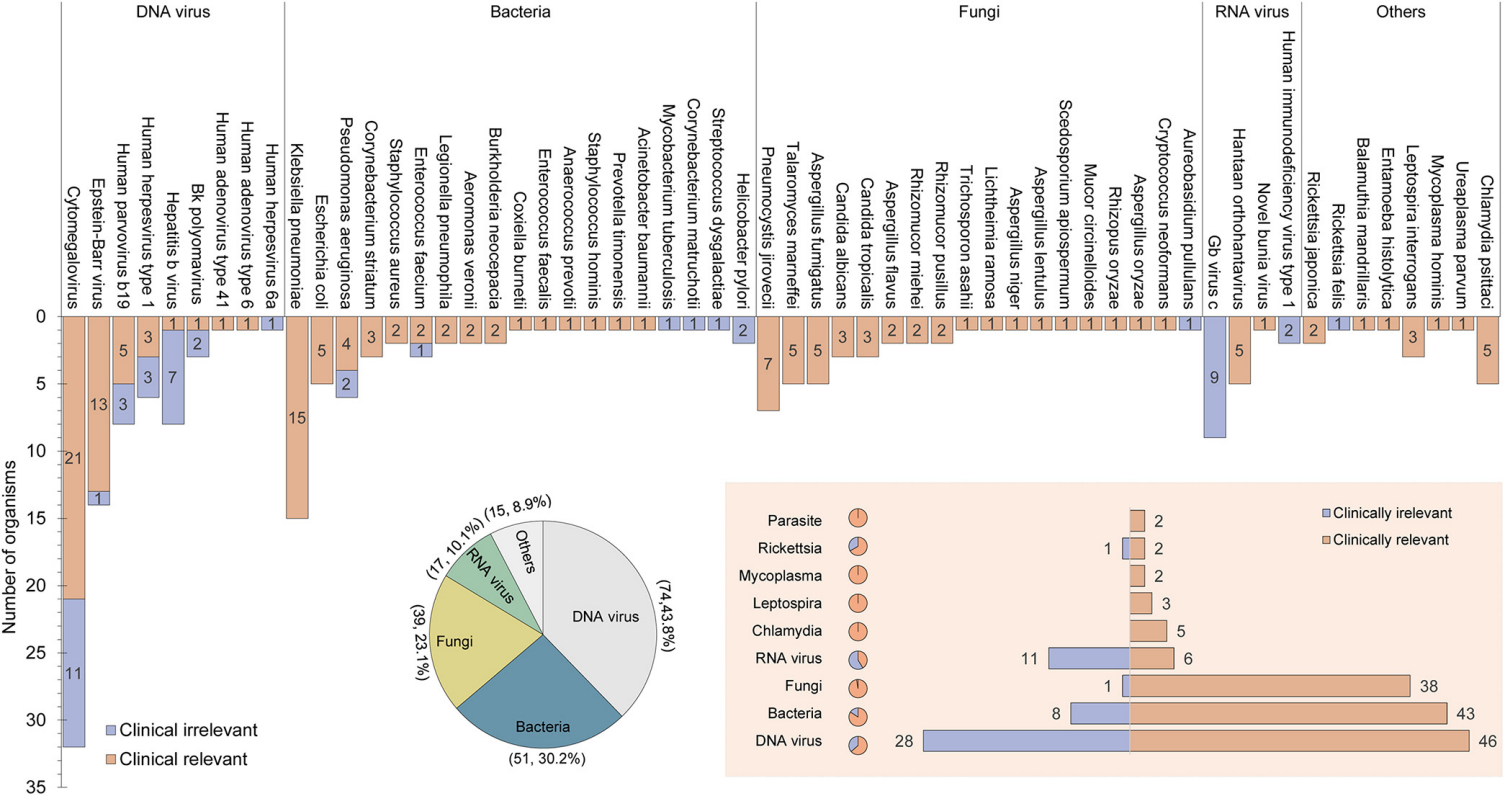
Summary of the proportions of organisms identified that resulted in clinical impact or were determined to be clinically irrelevant. The upper column chart shows all the organisms identified and reported, the lower pie chart depicts the proportion of organisms of different taxonomic groups, and the shaded bar chart in the lower right corner summarizes the total number of clinically relevant and unrelated organisms of different taxonomic groups.

A total of 196 organisms (58 species) were identified by plasma mNGS testing, of which more than 80% were bacteria (*n* = 51), fungi (*n* = 39), and DNA viruses (*n* = 74) (Fig. 2). A total of 75.6% (*n* = 148) of these organisms were finally considered clinically relevant by the treating team. Of the bacteria and fungi, only nine (eight bacteria, one fungus) were clinically irrelevant, including (i) two Helicobacter pylori that was thought to be associated with established chronic gastritis, and (ii) seven organisms (one Mycobacterium tuberculosis, two Pseudomonas aeruginosa, one Streptococcus dysgalactiae, one Corynebacterium matruchotii, one Enterococcus faecium, and one *Aureobasidium pullulans*) that might originate from lab contamination or normal human flora introduced during sampling or testing (see Table S2). By comparison, only 63.5% (47/74) of the detected DNA viruses were clinically relevant, which was lower than that of bacteria (84.3%, 43/51) and fungi (97.4%, 38/39) (Fig. 2). Cytomegalovirus (CMV), a human opportunistic pathogen, is the most common DNA virus identified, but only 65.6% (21/32) of positive mNGS tests were considered clinically relevant in this study. All the detection of Chlamydia, Mycoplasma, Leptospira, Rickettsia, and Parasites had significant clinical impacts on patient management except for Rickettsia felis, which was detected in a patient with suspected pulmonary infection but was not considered the causative agent, as the patient was finally diagnosed with pulmonary tuberculosis based on the positive sputum culture result for M. tuberculosis.

For the four reported RNA virus species, all five Hantaan orthohantavirus (HTNV) viruses with a low stringent mapped read number (SMRN) value (range, 1 to 19) were considered true pathogens because all the patients had a history of rat contact or mosquito bites and showed symptoms of epidemic hemorrhagic fever. The novel bunia virus with a high SMRN value (SMRN = 32,233) was responsible for the severe fever with thrombocytopenia syndrome (SFTS), subsequent multiple organ failure and rapid death of a 61-year-old female patient. All the detected GB C viruses (*n* = 9), even with a relatively high median SMRN value (median = 2,884), were thought to be transmitted by blood transfusion (all patients received frequent blood transfusions) with no clinical relevance. Two HIV viruses from AIDS patients were unrelated to the patient's current infections (see Table S2).

**FIG 3:**
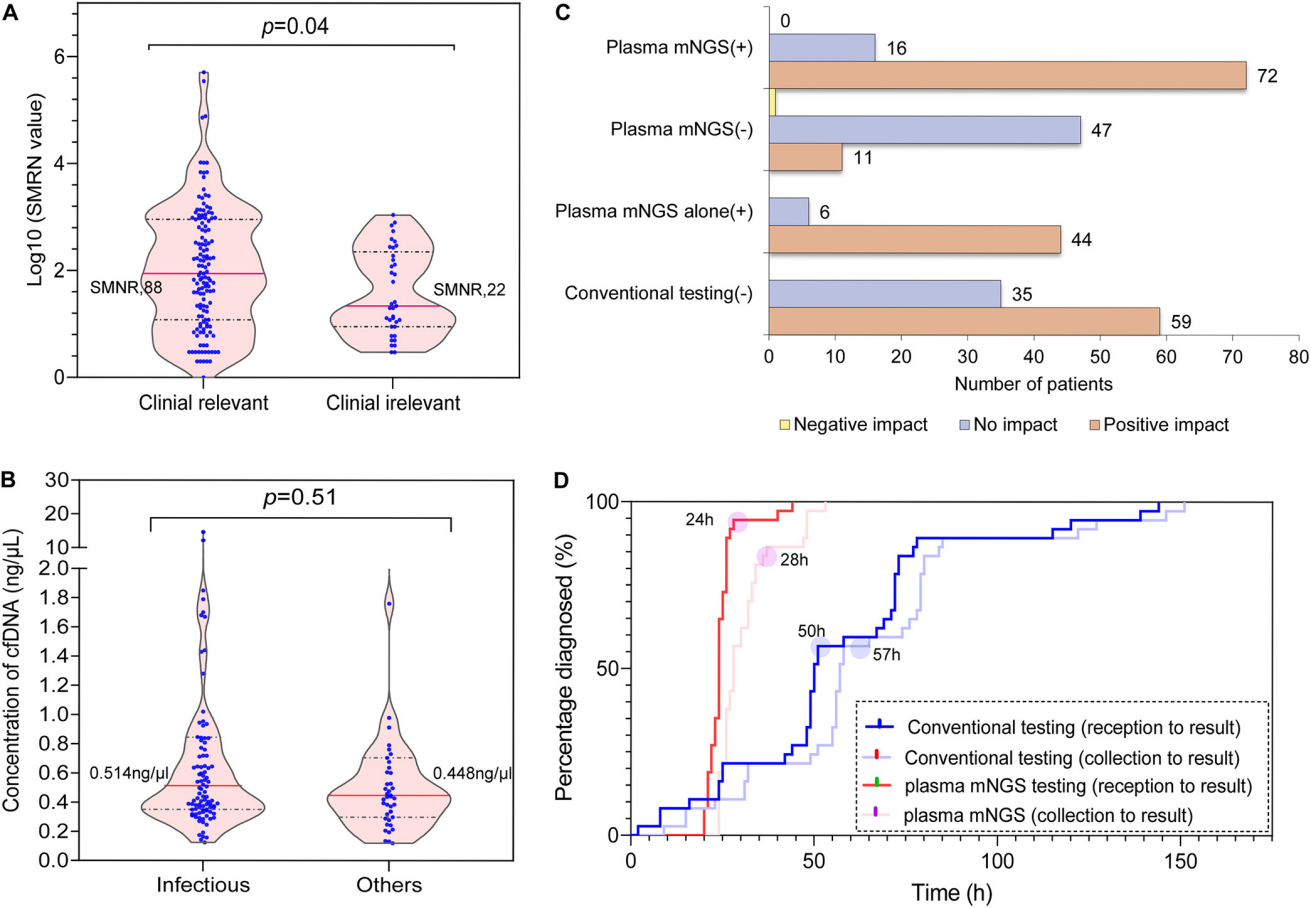
Clinical effect of mNGS testing. (A) The median stringent mapped read number (SMRN) of clinically relevant organisms was 88 versus 22 for irrelevant organisms. (B) The median cfDNA concentration in samples from the infectious patients was slightly higher than that in samples from other patients, but the difference was not statistically significant. (C) Clinical impact of plasma mNGS testing in subpopulations grouped based on different test results. Plasma mNGS (+)/(−) refers to patients with a positive/negative plasma mNGS result; plasma mNGS alone (+) refers to infectious patients identified with plasma mNGS alone; conventional testing (−) refers to patients with a negative conventional testing result. (D) The turnaround time of plasma mNGS testing in 37 patients was positive for both plasma mNGS and conventional testing.

We also assessed the relationship of the SMRN value of the above DNA organisms to the identification of a clinically relevant organism. The median SMRN value for clinically relevant organisms was 88 (interquartile range [IQR] = 12 to 907), in contrast to that of clinically irrelevant organisms (median RPM, 22; IQR = 9 to 230), which was a statistically significant difference (*P* = 0.04) (Fig. 3A). In addition, our study also found that the median cfDNA concentration in samples from the infectious patients was slightly higher than that from the other patients, although the difference was not statistically significant (median cfDNA concentration: 0.514 ng/μL versus 0.448 ng/μL, *P* = 0.51) (Fig. 3B).

Notably, the SMRN values of seven reported organisms from seven patients (two Chlamydia psittaci, one Aspergillus fumigatus, one *Rhizomucor pusillus*, Coxiella burnetii, one Entamoeba histolytica, and one HTNV virus) were below the preset threshold (SMRN = 3). These organisms were reported because the patients had clinical manifestations associated with their infection, and all patients improved rapidly after targeted treatment. This result underscores the importance of rigorous electronic medical record review and infectious disease consultation in the interpretation of mNGS results.

### Clinical impact of plasma mNGS testing on patient management.

**FIG 4:**
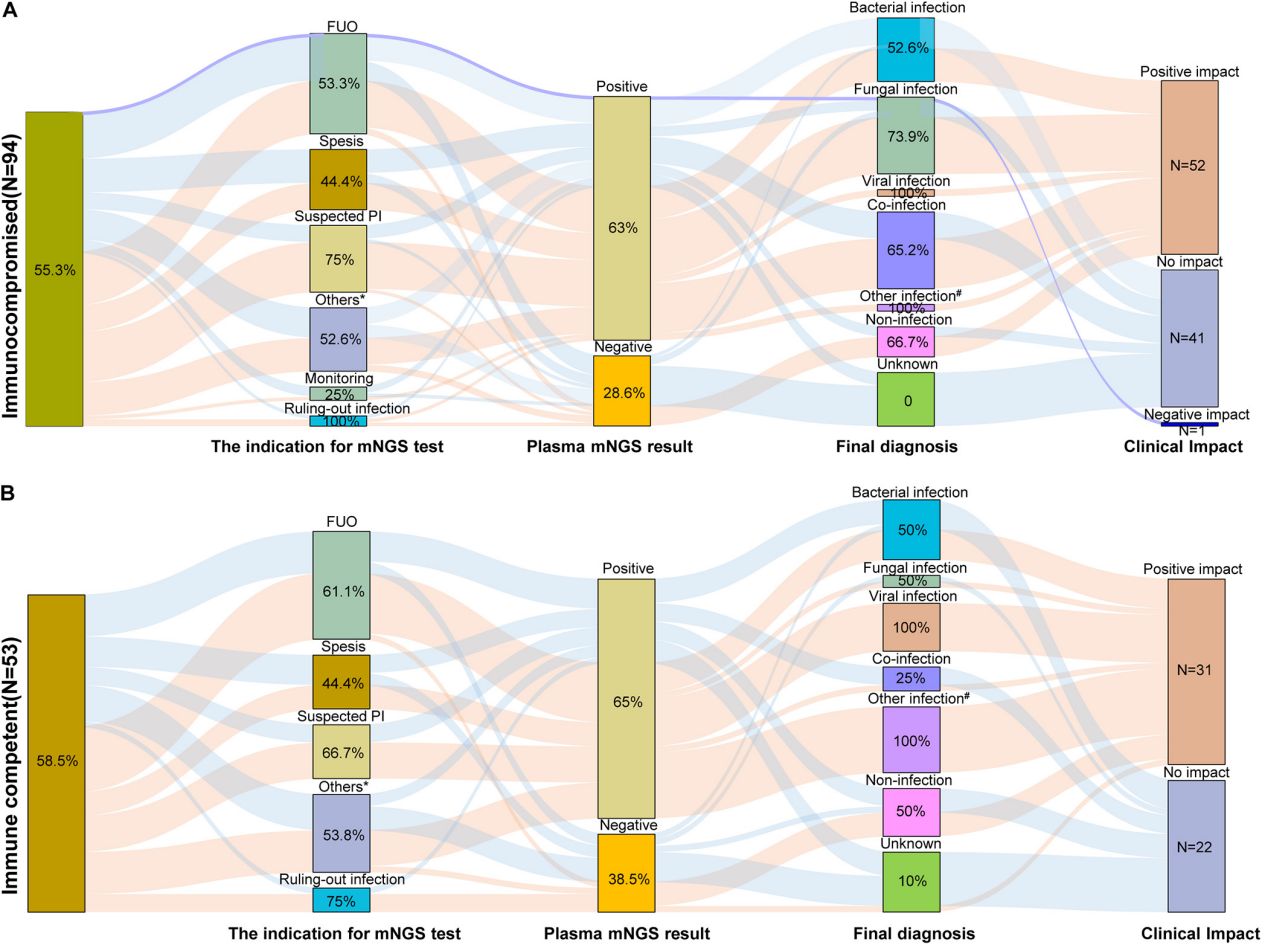
Summary of the clinical impact of plasma mNGS testing on patient management for immunosuppressed patients (panel A) and immunocompetent patients (panel B). *Includes suspected bloodstream infection without sepsis (SBI without sepsis), suspected abdominal infection (suspected AI), infective endocarditis (IE), suspected urinary tract infection (suspected UTI), suspected central nervous system infection (suspected CNSI) and abscess. ^#^Includes Chlamydia, Leptospira, richettsia, parasitic, and mycoplasma infection. The percentage shown in each column in the figure represents the proportion of plasma mNGS testing with positive clinical impact in each subgroup.

The plasma mNGS results led to a positive clinical impact in 83 (57.1%) patients (Table 3), involving 51.7% (15/29) of bacterial infections, 59.3% (16/27) of coinfections, 72% (18/25) of fungal infections, all other types of infections and 25.6% (11/43) of noninfections and undiagnosed cases (Fig. 4). A total of 62.8% (59/94) of the cases with negative results from conventional testing and 88% (44/50) of the infections identified with plasma mNGS alone showed a positive clinical impact on patient management (Fig. 3C). The positive clinical impact was categorized as enabling new diagnosis of infection not made by conventional methods (*n* = 47), enabling new diagnosis of infection earlier than conventional methods (*n* = 25), and facilitating ruling-out infection (*n* = 11) (Table 3; Table S2).

**TABLE 3:** Clinical impact of plasma mNGS testing (*n* = 147)

Categories of clinical impact	No. (%)of plasma mNGS Tests
Positive impact	83 (57.1%)
Enabled new diagnosis of infection and initiation of targeted therapy	32 (21.8%)
Enabled earlier diagnosis than conventional methods and initiation of targeted therapy	25 (17.0%)
Enabled new diagnosis of infection and escalation of therapy	8 (5.4%)
Enabled new diagnosis of infection and de-escalation of therapy	7 (4.8%)
Enabled ruling-out of Infection and initiation of noninfectious therapy	11 (7.5%)
No impact	63 (42.9%)
Redundant information, antibiotics and clinical plan were not changed	28 (19%)
A negative result with no clinical significance	16 (10.9%)
No relevant pathogen (considered transient unrelated bacteremia)	6 (4.1%)
No relevant pathogen (considered contamination)	6 (4.1%)
No relevant pathogen (considered pathogen associated with established chronic infection)	4 (2.7%)
Patient forgoes further treatment (discharge or death)	3 (2.0%)
Negative impact	1 (0.7%)
Lead to unnecessary treatment	1 (0.7%)

The impact was none in 63 (42.9%) patients (Table 3). Of these cases, 40 had microbes detected (i.e., positive plasma mNGS results), while 23 had no microbes detected (i.e., negative plasma mNGS results) (Fig. 3C). The lack of clinical impact of the 40 positive plasma mNGS results fell into three categories: (i) identification of a new organism (most commonly Klebsiella pneumoniae and some viruses) but no change in treatment strategy due to the effectiveness of empirical therapy (*n* = 21); (ii) the organisms were not considered to be the true pathogens by the treating team because the corresponding patients displayed no related clinical signs of infection (i.e., considered contamination from the environment or normal human flora, transient unrelated bacteremia or associated with established chronic infection) (*n* = 16); and (iii) identification of a new organism (Klebsiella pneumoniae, Escherichia coli, and *Aeromonas viridis*) but the patient forgoes further targeted therapy (*n* = 3) (see Table S2).

The only case associated with a negative clinical impact resulted in unnecessary antiviral therapy. This patient presented with fever of unknown origin during hospitalization for lymphoma. Cytomegalovirus (SMRN = 159) was identified by plasma mNGS testing and treatment with *foscarnet sodium* but was ineffective. The patient finally improved after switching to voriconazole based on the result of subsequent fecal culture that was positive for Candida parapsilosis (see Table S2).

### Turnaround time of plasma mNGS testing.

For samples that were positive for both plasma mNGS and conventional testing (*n* = 37), the estimated time-to-result for plasma mNGS was compared with conventional testing (Fig. 3D). The median turnaround time from sample collection to result for plasma mNGS was 28 h (IQR = 26 to 33.25), shorter than the median time to positive result for conventional testing (57 h; IQR = 54 to 79) (*P* < 0.01; Fig. 3D). The median time from receipt of the sample to positive result was 24 h (IQR, 22.75 to 26) for plasma mNGS compared with conventional testing (50 h; IQR = 47 to 72) (*P < *0.01; Fig. 3D). In addition, mNGS detected pathogens for the 37 patients on a median time of 2 days (IQR = 0.75 to 4.25) earlier than conventional testing (see Table S3). Although mNGS did not yet provide antimicrobial susceptibility testing like conventional microbial culture methods, this analysis demonstrates that the speed and with which species-level identification is provided by mNGS testing may offer significant benefit to patients.

## DISCUSSION

The potential clinical utility of plasma mNGS testing is likely to transform the field of diagnostic microbiology in the near future (2–4). However, most studies focus on the comparison of diagnostic performance with conventional methods, and few address the actual clinical impact on patient management of plasma mNGS tests in real-world clinical practice. In this study, we show that although plasma mNGS testing significantly improved the detection rate of tested samples, nearly one in four (24.5%, 48/196) mNGS tests reported organisms were not clinically relevant, emphasizing the importance of cautious interpretation and infectious disease consultation. Moreover, based on clinical adjudication, although only one plasma mNGS result resulted in a negative clinical impact, nearly half (42.9%, 63/147) of the plasma mNGS tests showed no impact for patient care and management in the current study, indicating that how best to integrate this advanced method into current infectious disease diagnostic frameworks to maximize its clinical utility in real-world practice is an important question. Our recent multicenter study showed that most current mNGS tests suffer from varying degrees of false positives and false negatives and that for a certain pathogen, the sensitivity of mNGS testing is not necessarily better than that of conventional microbiological methods (e.g., PCR) (16). These results are also supported by other studies (3, 17). Therefore, recommending plasma mNGS testing as a routine supplement to first-line diagnostic tests for infectious diseases faces great challenges (18, 19). We agree with Hogan et al. that the decision to conduct mNGS testing should take into account the diagnostic performance, turnaround time, and cost-effectiveness of mNGS, as well as the availability of conventional tests (5).

Cell-free microbial nucleic acid fragments in plasma may originate from infection sites or nonpathogenic colonizing organisms (4). Distinguishing the role (infection or colonization) of the identified organisms in patients is a critical challenge for plasma mNGS testing, especially in immunocompromised hosts and patients with compromised mucosal barrier functions. Due to differences in sample handling, pathogenicity of viral genotypes and microbial strains, underlying conditions of patients, and individualized treatment, it is almost impossible to establish universal diagnostic criteria for determining whether an organism causes an infection based on the detected sequences. We found that the median SMRN value of clinically relevant organisms was higher than that of unrelated organisms (88 versus 22, *P* = 0.04), but this was not a threshold for a certain case. In most cases, clinicians still need to make empirical diagnoses and actions based on their professional knowledge, as they do when interpreting the results from routine microbiological cultures (20). For example, seven patients in this study had organisms (Chlamydia psittaci, Aspergillus fumigatus, *Rhizomucor pusillus*, Coxiella burnetii, Entamoeba histolytica, and *HTNV virus*) detected in samples with SMRN values below the reporting threshold (SMRN = 3), but these organisms were clinically considered to be true pathogens and targeted therapy was initiated. Here, we must emphasize that the reporting of these subthreshold results based on clinical chart review is not a standard approach and is not feasible in many situations. We recommend that for organisms that do not meet the reporting threshold but are consistent with clinical manifestations, it is necessary to use additional microbiological methods, such as culture, PCR, or sanger sequencing, to verify mNGS results to increase the reliability of clinical prediction.

Moreover, mNGS is currently unable to provide reliable data on antimicrobial sensitivity and resistance, suggesting that the professional ability of the result interpretation team, especially infectious disease expertise, is directly related to the clinical utility of mNGS testing (21). Establishing a clinical microbial sequencing board or team composed of experienced clinical microbiologists, infectious disease specialists, and treating physicians has been strongly recommended to avoid misinterpretation of mNGS results (22). Our laboratory has established a stable reporting team for the mNGS assay (described in the Supplementary Methods) and carefully interprets each result through close communication with the treating team. This may be one of the reasons why the clinical relevance of the reported organism (75.6%) is higher compared to previous studies (6, 23).

The finding that 56.5% of plasma mNGS test results in this study led to positive clinical impact is similar to that reported by Rossoff et al., where 56 of 100 (56.0%) test results were considered clinically actionable (21), but in contrast to that recently reported by three retrospective studies (7.3% to 26%) (5, 6, 8). Patient selection practices may have contributed to the higher positive clinical impact in this study. As described in the Introduction section, most plasma mNGS tests were performed in this study when conventional methods were negative, useful invasive samples that could not be obtained or the patient was too sick to wait for conventional test results with a long turnaround time. In such cases, the treating team would first consider the relatively rapid mNGS results (approximately 24 h) as important evidence for an earlier diagnosis. However, in the subset of 34 negative mNGS tests in our clinical practice, only 32.4% (11 tests) showed a positive clinical impact. Five tests failed to detect the pathogens associated with local or deep-seated infection (i.e., Candida albicans in two suspected PIs, Enterococcus faecium in one suspected UTI, *Nocardia* in a chest wall abscess and *Kodamaea ohmeri* in one suspected AI), and 18 tests did not lead the treating team to initiate a decision to withdraw antimicrobials (see Table S2). These results suggest the conclusion that plasma mNGS testing may not be a reliable tool to rule out infection in complex patients (3, 5, 6, 24). Taken together with other studies, all authors tend to believe that the ideal mNGS testing would be used in those patients with a high probability of being positive and impactful (10). Furthermore, in contrast to the study by Lee et al., the present study did not find plasma mNGS testing to be more valuable for immunocompromised patients than for immunocompetent patients (55.3% versus 58.5%, *P* = 0.73) (Table 1; Fig. 4). This inconsistent conclusion needs to be confirmed by future studies. Undoubtedly, mNGS has absolute advantages in diagnosing rare clinical infections or identifying rare pathogens with public health impact, such as HTNV, HIV, novel bunia virus, Rickettsia, Balamuthia mandrillaris, Entamoeba histolytica, Leptospira interrogans, and Chlamydia psittaci identified in this study, which not only provide very important etiological evidence for patients to obtain accurate and timely diagnosis, but also, more importantly, can play a very important role in in preventing the spread of pathogens in communities or hospitals.

This study first assessed the utility of plasma mNGS testing in the detection of RNA viruses. The HTNV and novel bunia virus found in the fever of unknown origin (FUO) patients with a history of rat contact or mosquito bites provided timely pathogenic evidence for the timely treatment of patients. However, most of the infections that occur in hospitalized patients are caused by DNA pathogens, so whether it is necessary to carry out RNA virus detection for each patient in a cost-increasing manner is a question worth considering. Other issues in clinical practice, such as whether the discovery of an organism with unclear clinical pathogenicity (e.g., GB C virus) or associated with known chronic infection (e.g., HIV), must be reported to the treating team also requires further discussion. Ultimately, as with any diagnostic tool, result interpretation is greatly influenced by pretest probability. How best to identify these patients, although, remains a challenge.

There are limitations to this study. First, due to the clinical complexity of patients, it is difficult to find a fair gold standard to standardize the definition of clinical impact criteria. This problem also exists in previous studies (5, 6). We assessed the clinical impact in this study based on whether the availability of a test could have changed patient management and the subsequent patient outcome. This may have over- or underestimated the actual impact of the plasma mNGS testing result. Second, the patients enrolled in this study came from multiple ordering providers and involved multiple disease types. Due to the limited number of cases, we did not perform a stratified analysis. We will discuss the clinical utility of mNGS testing in specific populations (e.g., sepsis, infective endocarditis) in further studies. Third, because most of the patients were treated with empirical antimicrobial drugs because of critical illness, we were unable to compare the clinical value of mNGS in empirically treated and untreated populations.

In summary, despite the insights gained in our study regarding the diagnostic performance and clinical utility of plasma mNGS testing in real-world use, for such a complex microbial detection technology, only through close communication between the laboratory and the clinic can we accurately screen clinically meaningful results for patients from a large amount of data generated by mNGS. More clinical practice and studies are needed to guide patient selection, timing of testing initiation, and cautious clinical interpretation.

## MATERIALS AND METHODS

### Study population and ethical considerations.

A total of 166 patients underwent plasma mNGS testing in either an inpatient or outpatient setting at the First Affiliated Hospital, Zhejiang University School of Medicine (FAHZU) between February and October 2021 (Fig. 1A). We excluded 10 patients who did not have sufficient sample size or whose mNGS tests failed and nine patients who could not be followed up. The remaining 147 cases were enrolled in this study to assess the diagnostic performance and clinical impact of plasma mNGS testing for DNA- and RNA-based pathogens. Similar to previous studies (7, 24), the etiologic diagnosis of each patient was determined by the clinical treatment team based on all microbiological tests (including cultures, serology, PCR, and mNGS), other laboratory tests (e.g., microbiological, biochemical, immunological, hematological, and oncological assays), radiology results, clinical manifestations, treatment, and disease outcomes. This study was approved by the FAHZU institutional review board (IIT20220714A).

### Plasma mNGS testing.

The testing protocol, experimental parameters, quality control, and test result reporting of the plasma mNGS testing in our laboratory are described in the Supplementary Methods. The microorganisms that can be detected by plasma mNGS testing are also listed in the Supplementary Appendix (see in Table S1). DNA and RNA libraries that were generated from blood samples obtained from patients were each sequenced to a mean depth of 20 million. We defined a normalized bioinformatics parameter, SMRN, to represent the number of reads per organism at the sequencing depth of 20 million. SMRN was calculated as below:

$$\mathrm{SMRN}=\frac{20\;\mathrm{milions}\times \mathrm{Number}\;\mathrm{of}\;\mathrm{reads}\;\mathrm{only}\;\mathrm{mapped}\;\mathrm{within}\;\mathrm{same}\;\mathrm{taxon}}{\mathrm{Total}\;\mathrm{reads}\;\mathrm{of}\;\mathrm{this}\;\mathrm{sample}}$$

The detected microbes and their SMRNs were sent to providers (or treating team) by a reporting team in the laboratory (see Supplementary Methods).

### Evaluation of the clinical utility of plasma mNGS testing.

**(i) Diagnostic performance.** In this study, we calculated the diagnostic performance of plasma mNGS testing (each test was interpreted as a whole) using a method similar to “method 1” employed to evaluate the test performance characteristics for plasma cfDNA mNGS findings in Lee et al. (6) The etiologic diagnosis of each patient determined by the clinical treating team was used as a composite reference standard to calculate the positive and negative percent agreements (PPA and NPA) of plasma mNGS by test sent. In infectious cases, a positive plasma mNGS test was considered true positive (TP); a negative plasma mNGS test was defined as false negative (FN). In the remaining patients, a positive mNGS test was considered a false positive (FP), and a negative mNGS test was considered a true negative (TN) (Table 2).

**(ii) Clinical relevance of organisms identified with plasma mNGS testing.** We determined the clinical relevance of each organism by reviewing the treating team's responses to them. Clinically relevant organisms included: (i) consistent with the results of all conventional microbiological tests (culture, serology, and/or PCR) performed within 7 days of presentation and determined by the treatment team to be a true pathogen; and (ii) inconsistent with the conventional microbiological tests, but the treatment team trusted mNGS and implemented targeted treatment. Clinically irrelevant organism refers to: (i) insufficient clinical evidence related to its infection and targeted treatment was not initiated; and (ii) considered contamination from the environment or normal human flora, transient unrelated bacteremia, or associated with established chronic infection. All of the above information comes from the review of each patient's electronic medical record (EMR) and clinical consultation with an ordering clinician or treating team.

**(iii) Clinical impact of plasma mNGS testing.** In our hospital, every mNGS result was reported timely to the treating team and prospectively used for clinical management. The treating team’s interpretation of plasma mNGS results and their subsequent management decisions and actions were considered to assess the real-world impact of plasma mNGS testing. Specifically, whether the mNGS test result has a positive, negative, or no clinical impact was determined primarily based on (i) whether real-time availability of plasma mNGS testing could have changed the clinical reasoning, management of patient care (e.g., whether antimicrobials were added or changed), or both; and (ii) the subsequent patient response (i.e., improved, delayed, worsened, or died). The specific categories of each type of clinical impact are shown in Table 3. In addition, descriptive statistical analyses of the result time and TAT between plasma mNGS testing and conventional testing were performed.

### Statistical analysis.

Demographic data were summarized using descriptive statistics. The statistical significance of differences in stringent mapped read number (SMRN) between clinically relevant and irrelevant organisms, cfDNA concentration between infectious and other patients, and TAT between plasma mNGS testing and conventional testing was analyzed using the Mann–Whitney test for group medians, assuming that observations were not normally distributed. Statistical tests were performed using SPSS 18 software (SPSS Inc., Chicago IL, USA). and GraphPad version 8.0.1 software (GraphPad Software, San Diego, CA, USA) with a *P* value ≤ 0.05 as the significance threshold.

### Data availability.

The pathogen reads of our study were deposited in the Genome Warehouse in the National Genomics Data Center (National Genomics Data Center Members and Partners, 2021) under project PRJCA014111, which are publicly accessible at https://bigd.big.ac.cn/gsa.
